# Development and mixed-methods evaluation of an online animation for young people about genome sequencing

**DOI:** 10.1038/s41431-019-0564-5

**Published:** 2020-01-02

**Authors:** Celine Lewis, Saskia C. Sanderson, Jennifer Hammond, Melissa Hill, Beverly Searle, Amy Hunter, Christine Patch, Lyn S. Chitty

**Affiliations:** 1 London North Genomic Laboratory Hub, Great Ormond Street Hospital, London, UK; 2 UCL Great Ormond Street Institute of Child Health, London, UK; 3 Department of Behavioural Science and Health, University College London, London, UK; 4 Unique—The Rare Chromosome Disorder Support Group, Oxted, UK; 5 Genetic Alliance UK, London, UK; 6 Genomics England, Queen Mary University of London, Dawson Hall, London, UK; 7 Faculty of Health and Wellbeing, Sheffield Hallam University, London, UK; 8 Counselling, Society and Ethics Research, Wellcome Genome Campus, Cambridge, UK

**Keywords:** Social sciences, Genetics

## Abstract

Children and young people with rare and inherited diseases will be significant beneficiaries of genome sequencing. However, most educational resources are developed for adults. To address this gap in informational resources, we have co-designed, developed and evaluated an educational resource about genome sequencing for young people. The first animation explains what a genome is, genomic variation and genome sequencing (“My Genome Sequence”: http://bit.ly/mygenomesequence), the second focuses on the limitations and uncertainties of genome sequencing (“My Genome Sequence part 2”: http://bit.ly/mygenomesequence2). In total, 554 school pupils (11–15 years) took part in the quantitative evaluation. Mean objective knowledge increased from before to after watching one or both animations (4.24 vs 7.60 respectively; *t* = 32.16, *p* < 0.001). Self-rated awareness and understanding of the words ‘genome’ and ‘genome sequencing’ increased significantly after watching the animation. Most pupils felt they understood the benefits of sequencing after watching one (75.4%) or both animations (76.6%). Only 17.3% felt they understood the limitations and uncertainties after watching the first, however this was higher among those watching both (58.5%, *p* < 0.001). Twelve young people, 14 parents and 3 health professionals consenting in the 100,000 Genomes Project reported that the animation was clear and engaging, eased concerns about the process and empowered young people to take an active role in decision-making. To increase accessibility, subtitles in other languages could be added, and the script could be made available in a leaflet format for those that do not have internet access. Future research could focus on formally evaluating the animations in a clinical setting.

## Introduction

Children and young people will be significant beneficiaries of genome sequencing (GS) technology as the majority (50–75%) of rare diseases affect children [1]. It is considered a good practice to involve young people in decisions about their care and treatment even if legally they are unable to provide informed consent. A recent statement from the American College of Medical Genetics and Genomics (ACMG) advocated “a robust engagement process with the mature older child and adolescent patient to facilitate meaningful conversation that can aid in the complex decision-making and return of findings process around genomic testing” [2].

In recent years, a number of online educational resources about GS have been developed for patients. However, these are primarily aimed at adults [3], have been developed for people taking part in specific projects such as the 100,000 Genomes Project [4, 5], or are not designed for use in the clinic [6]. A gap exists in terms of information resources about GS that have been developed specifically for young people. Young people with health-related issues are likely to face physical, psychological and social challenges that differ significantly from those of both children and adults [7, 8]. As such it is seen as important to involve young people in the development of interventions designed for their use [9]. Moreover, information needs and preferred communication formats differ between children and adults [10].

The use of animations has been shown to be an effective method for educating children and students [11–13]. Studies have shown that, particularly in biology, students who learn with animations compared with traditional lectures obtain significantly higher marks [14], and that animations are more effective than static sequential images [15]. Research has also shown that students who use animations as supplementary learning materials are more interested in the subject, and suggests that animations are particularly useful for learning complicated concepts [16]. Further benefits of animations are that they can offer some degree of control over learning pace (the ability to pause, play, rewind) and can be watched at a time that is most suitable to the viewer [17]. Young people also regularly access online content: In the UK in 2016, 98% of children and adolescents had access to the internet, 83% of 12- to 15-year-olds had their own smartphone and 55% had their own tablet [18].

In preparation for the implementation of GS as a clinical service in the UK National Health Service (NHS), we developed and evaluated two animations that were co-designed with young people. Our primary aim was to develop an educational resource that explained GS for young people accessing diagnostic testing for rare diseases, however, we were also keen that it would be a useful learning resource for young people more generally. The target age group for animations were 11–15 years. We focused on this age group as in the 100,000 Genomes Project, 11–15 year olds are encouraged to be active participants in the decision-making process and sign an ‘assent’ form if they would like to take part [19]. It is likely that this approach will be adopted into clinical practice.

Here we report on (1) the development, and (2) evaluation of the animations in terms of knowledge, attitude and satisfaction.

## Materials and methods

### Guiding theory

The development of the animation was guided by the cognitive theory of multimedia learning which predicts that multimedia presentations, such as narrated animation, are more likely to lead to meaningful learning than single-medium presentations [20]. The theory includes a collection of research-based principles for the design of animations. These include that (1) students learn more deeply when corresponding portions of the narration and animation are presented at the same time than when they are separated in time, (2) that they learn more deeply from animation and narration than from animation and on-screen text, (3) that they learn more deeply when narration is in conversational rather than formal style, and (4) that only a few pieces of information can be actively processed at any one time (limited capacity assumption).

### Development of the animation

The animation was co-designed with (1) young people taking part in the 100,000 Genomes Project, (2) school pupils, (3) members of a children’s hospital Young Person’s Advisory Group and (4) members of an expert working group. The design process consisted of three phases; (1) content development, (2) script and story development, and (3) animation development. The first animation (‘My Genome Sequence part 1’) explains what a genome is and how variations in the genome cause different conditions, and what genome sequencing is. The second (‘My Genome Sequence part 2’) focusses on the limitations and uncertainties of the technology. Development of both animations included extensive engagement with young people who co-designed the script including use of words, selected the images used and delivered the voice over. For an in-depth account of the development process, refer to supplementary material (Supplementary Materials 1–4).

### Mixed-methods evaluation

#### Study design

##### Quantitative evaluation

School pupils were recruited to take part in a survey study (Supplementary Material 5) using a before and after study design to quantitatively evaluate the effectiveness of the animation as an educational tool.

##### Qualitative evaluation

Young people were shown the first animation during the 100,000 Genomes Project consent appointment and follow-up interviews were then conducted with the young person, their parents and the health professional.

#### Recruitment

##### Quantitative evaluation

An opportunistic recruitment method was used to identify schools that might potentially be interested in taking part in the evaluation study. Seven head teachers or head of science teachers known to CL and located in schools in London, the South East of England and the South West of England were contacted via email. The email explained the aims of the study and included a link to the animation(s) and a copy of the survey. Staff from six schools agreed to take part in the evaluation study. The evaluation occurred in two stages. In July 2017 we evaluated the first animation (“Study 1”) in two schools (Secondary 1 and Secondary 2). Secondary 1 was located in Hackney, London; the school catchment area has an Index of Multiple Deprivation Decile (IMD) of 4 out of 10 (where 1 is most deprived), meaning that the school is located in one of the most deprived 40% of areas in England. Secondary 2 was in Southend-On-Sea (IMD of 7). Between March and July 2018 we evaluated both animations (“Study 2”) in four schools (Primary 1, Primary 2, Secondary 3 and Secondary 4). Primary 1 was located in Islington, London (IMD of 7), Primary 2 was located in Wiltshire (IMD of 8), Secondary 3 and 4 were both located in Hackney, London (both have an IMD of 4).

##### Qualitative evaluation

The first animation was shown to young people aged 11–15 years and their families at a large children’s hospital in London, in August 2018, during the last month of recruitment into the 100,000 Genomes Project. We decided only to show the first as we were unsure how well the animation would be received during the consent appointment. Young people were excluded if they had moderate to severe learning difficulties (this was based on prior medical knowledge of the patient) or if they and/or their parents did not speak English and were thus unable to consent or assent to take part. At the end of the consultation, they were invited to take part in a face-to-face 15 minute semi-structured interview that took place whilst they were still in the consultation room or in the phlebotomy waiting room (conducted by CL). Once recruitment had closed, staff members consenting families into the 100,000 Genomes Project (‘consenters’), who had shown the animation during the appointment were also invited to take part in a face-to-face semi-structured interview.

Young people and their parents were asked: what they thought of the animation, what impact it had on the consent appointment, whether it had an impact on their understanding of genome sequencing and at what point it would be most useful to have seen the animation. Health professionals were asked: what impact the animation had on the consultation and at what point they thought it was most effective to show the animation. All interviews were audio-recorded and transcribed verbatim.

#### Survey development

Participants completed a quantitative survey which was implemented at two time-points; time 1 (T1) before watching the animation and time 2 (T2) after watching the animation (Supplementary Material 5). The survey was adapted from one developed by Sanderson et al. [21] to evaluate the animation “Whole Genome Sequencing and You” [3] and included a new knowledge of genome sequencing measure for young people (the kids-KOGS) [22].

The T1 survey included (1) participant characteristics (age and gender), (2) subjective knowledge and (3) objective knowledge. The T2 survey included (1) subjective knowledge, (2) objective knowledge, (3) questions to explore attitude towards GS including intention to undergo GS, and (4) questions to explore satisfaction with the animation.

To ensure the survey was appropriate for an 11–15 year age group, the survey was piloted using think-aloud interviews, with two young people taking part in the 100,000 Genomes Project, two science teachers and one adult parent. Participants were asked to verbalise their thoughts as they read the questions in the survey, giving feedback on wording, comprehension and ease of answering. Following this process, revisions were made in light of feedback. The survey was then piloted with pupils in one primary school (83 pupils aged 11) at which point some minor changes were made to the wording.

##### Knowledge

The survey included measures to assess self-rated and objective knowledge. *Self-rated knowledge* was assessed using five key terms: DNA, gene, chromosome, genome, genome sequencing. For each term participants were asked “have you heard this word before” (Yes or No), and “Do you know what this word means” (Yes or No). Responders were also asked “How would you describe your understanding of genetics” (None, Some, Good). *Objective knowledge* was assessed using the new 10-item kids-KOGS [22] which includes a series of statements about GS with responders asked to indicate whether the statement is true, false or don’t know.

##### Attitude

The survey included seven questions to examine attitude towards GS. These included examining whether responders “understand the benefits of genome sequencing”, “understand the limitations of genome sequencing” and whether, if they had a health problem and the doctor suggested GS they would want to have it. For each question, multiple answer options were available e.g. agree, disagree, not sure.

##### Satisfaction

Satisfaction with the animation was assessed using six questions about understandability, amount, length, look and impact. Each question had multiple choice answer points e.g. too much, too little, the right amount.

### Analysis

#### Quantitative evaluation

Descriptive statistics were used for participant characteristics. For objective knowledge, missing data were treated as ‘Don’t know’ and scored as incorrect. The differences between Study 1 and 2 were tested with non-parametric statistics (Wilcoxon Signed Ranks Test). Between-group differences were compared with Pearson *X*^2^ test. Logistic regression models were used to test the impact of demographic characteristics on outcome variables. All tests are two-sided and a *p* value <0.05 was considered significant.

#### Qualitative evaluation

Transcripts were analysed using thematic analysis [23] This process involved (1) familiarising with the data, (2) general initial codes, (3) searching for themes, (4) reviewing themes, and (5) defining and naming themes. Analysis was conducted by two researchers (JH and CL) to ensure trustworthiness.

## Results

### Quantitative evaluation

An anonymous paper survey was administered to 554 pupils aged 11–15 years (289 in Study 1 and 265 in Study 2). Sample characteristics are provided in Table 1. The mean age was 12.8 years; 59.7% were female. There was a significant difference in mean age in Study 1 and Study 2 (13.21 and 12.43 respectively, *p* < 0.001) and gender (52.6% female and 67.8% female respectively, *p* < 0.001).

**Table 1:** Participant characteristics.

Characteristic	% (*n*)
**Age, years**	
Mean (SD)	12.8 (1.3)
11	17.0% (94)
12	30.5% (169)
13	18.9% (103)
14	19.9% (110)
15	14.1% (78)
**Gender**	
Female	331 (59.7%)
Male	221 (39.9%)
**School**	
Primary 1	3.4% (19)
Primary 2	6.7% (37)
Secondary 1	27.4% (152)
Secondary 2	24.7% (137)
Secondary 3	17.7% (98)
Secondary 4	20.0% (111)

In some cases there is missing data, so numbers may not add up to total *N* (554)

### Knowledge

Self-rated understanding of genetics significantly increased in both Study 1 and Study 2 (Table 2). Self-rated awareness and self-rated understanding of the words ‘genome’ and ‘genome sequencing’ increased significantly after watching the animation in both Study 1 and Study 2.

**Table 2:** Subjective knowledge.

	Study 1; *N* = 289			Study 2; *N* = 265		
Measure	Pre	Post	Test statistics, *P* value	Pre	Post	Test statistic, *P* value
None	42 (14.5%)	14 (4.8%)	*Z* = −7.98, ***p*** = 1.43^**−15**^	46 (17.4%)	24 (9.1%)	*Z* = −8.42, ***p*** = 3.80^**−17**^
Some	208 (72.0%)	185 (64.0%)		174 (65.7%)	122 (46.0%)	
Good	35 (12.1%)	84 (29.1%)		40 (15.1%)	113 (42.6%)	
Heard the word DNA:						
Yes	288 (99.7%)	286 (99.0%)	*Z* = 0.00, *p* = 1.00	261 (98.5%)	261 (98.5%)	*Z* = −1.73, *p* = 0.08
No	1 (0.3%)	1 (0.3%)		3 (1.1%)	0 (0.0%)	
Heard the word gene:						
Yes	287 (99.3%)	284 (98.3%)	*Z* = −1.00, *p* = 0.32	237 (89.4%)	242 (91.3%)	*Z* = −2.32, ***p*** **=** **0.02**
No	2 (0.7%)	3 (1.0%)		27 (10.2%)	18 (6.8%)	
Heard the word chromosome:						
Yes	250 (86.5%)	246 (85.1%)	*Z* = −0.33, *p* = 0.74	194 (73.2%)	207 (78.1%)	*Z* = −3.41, ***p*** = 0.001
No	38 (13.1%)	38 (13.1%)		69 (26.0%)	53 (20.0%)	
Heard the word genome:						
Yes	100 (34.6%)	260 (90.0%)	*Z* = −12.65, ***p*** = 1.13^**−36**^	82 (30.9%)	231 (87.2%)	*Z* = −12.09, ***p*** = 1.23^**−33**^
No	185 (64.0%)	25 (8.7%)		181 (68.3%)	28 (10.6%)	
Heard the word genome sequencing:						
Yes	68 (23.5%)	269 (93.1%)	*Z* = −14.07, ***p*** = 5.71^**−45**^	45 (17.0%)	238 (89.8%)	*Z* = 13.68, ***p*** = 1.31^**−42**^
No	216 (74.7%)	16 (5.5%)		218 (82.3%)	22 (8.3%)	
Know what DNA means:						
Yes	269 (93.1%)	279 (96.5%)	*Z* = −3.21, ***p*** = 0.001	241 (90.9%)	252 (95.1%)	*Z* = −2.98, ***p*** = 0.003
No	20 (6.9%)	8 (2.8%)		23 (8.7%)	3.4 (98.5%)	
Know what gene means:						
Yes	267 (92.4%)	276 (95.5%)	*Z* = −3.16, ***p*** = 0.002	216 (81.5%)	231 (87.2%)	*Z* = −3.00, ***p*** = 0.003
No	20 (6.9%)	10 (3.5%)		48 (18.1%)	29 (10.9%)	
Know what chromosome means:						
Yes	190 (65.7%)	197 (68.2%)	*Z* = −2.31, *p* = 0.021	136 (51.3%)	155 (58.5%)	*Z* = −4.04, ***p*** = 0.000053
No	94 (32.5%)	83 (28.7%)		127 (47.9%)	105 (39.6%)	
Know what genome means:						
Yes	29 (10.0%)	209 (72.3%)	*Z* = −13.42, ***p*** = 4.86^**−41**^	21 (7.9%)	188 (70.9%)	*Z* = −12.81, ***p*** = 1.44^**−37**^
No	258 (89.3%)	71 (24.6%)		241(90.9%)	72 (27.2%)	
Know what genome sequencing means:						
Yes	24 (8.3%)	253 (87.9%)	*Z* = −15.03, ***p*** = 4.41^**−51**^	17 (6.4%)	222 (83.8%)	*Z* = −14.25, ***p*** = 4.58^**−46**^
No	262 (90.7%)	29 (10.0%)		245 (92.5%)	38 (14.3%)	

In some cases there is missing data, therefore numbers may not add up to 289 or 265 (100%). Subjective knowledge scores were compared using the Wilcoxon Signed Ranks Test

Note: *p* values in bold indicate significance over 0.05

Objective knowledge increased significantly (*p* < 0.05) after watching the animation for *all* the knowledge items for both studies (Table 3). Notably, a greater proportion of participants in Study 2 answered Item 10 correctly (on the limitations and uncertainties of genome sequencing) post-intervention compared to participants in Study 1 (Study 1: 33.9%, *Z* = −3.17, *p* = 0.002; Study 2: 54.0%, *Z* = −7.49, *p* = 6.97^−14^).

**Table 3:** Objective knowledge using 10-item kids-KOGS.

	Study 1; *N* = 289			Study 2; *N* = 265		
Item	Pre	Post	Sig.	Pre	Post	Sig.
Genetics 101						
**Item 1:** Our DNA is inside our cells (true)	248 (85.8%)	260 (90.0%)	*Z* = −2.69, ***p*** = 0.007	213 (80.4%)	238 (89.8%)	*Z* = −3.703, ***p*** = 0.000213
**Item 2:** Our DNA doesn’t have an effect on how our body works (false)	206 (71.3%)	241 (83.4%)	*Z* = −4.52, ***p*** = 0.000006	178 (67.2%)	207 (78.1%)	*Z* = −3.54, ***p*** = 0.000396
**Item 3:** Our complete set of DNA is called our genome (true)	53 (18.3%)	254 (84.8%)	*Z* = −13.68, ***p*** = 1.38^**−42**^	70 (26.4%)	207 (78.1%)	*Z* = −11.33, ***p*** = 9.97^**−30**^
**Item 4:** Around 1% of our genome is the same as other people’s (false)	39 (13.5%)	212 (73.4%)	*Z* = −12.65, ***p*** = 1.10^**−36**^	38 (14.3%)	167 (63.0%)	*Z* = −10.17, ***p*** = 2.79^**−24**^
**Item 5:** Our genome is more similar to our close relatives, like our mum and dad, than it is with other people’s (true)	150 (51.9%)	247 (85.5%)	*Z* = −9.05, ***p*** = 1.50−^**19**^	173 (65.3%)	237 (89.4%)	*Z* = −6.75, ***p*** = 1.52^**−11**^
**Item 7:** A ‘glitch’ in the genome (like a spelling mistake) can cause a health problem because the body isn’t getting the right instructions (true)	117 (40.5%)	274 (94.8%)	*Z* = −12.53, ***p*** = 5.12^**−36**^	134 (50.6%)	232 (87.5%)	*Z* = −9.34, ***p*** = 9.28^**−21**^
Genome sequencing						
**Item 6:** Genome sequencing involves looking at all the DNA in a person’s genome (true)	66 (22.8%)	189 (65.4%)	*Z* = −9.98, ***p*** = 1.94^**−23**^	59 (22.3%)	170 (64.2%)	*Z* = −9.63, ***p*** = 6.28^**−22**^
**Item 8:** Genome sequencing can be done on the DNA in a blood sample (true)	71 (24.6%)	265 (91.7%)	*Z* = −13.68, ***p*** = 1.31^**−42**^	74 (27.9%)	232 (87.5%)	*Z* = −10.19, ***p*** = 2.22^**−24**^
Limitations and uncertainties						
**Item 9:** We know all there is to know about what our genome does (false)	174 (60.2%)	213 (73.7%)	*Z* = −4.67, ***p*** = 0.000003	153 (57.7%)	182 (68.7%)	*Z* = −3.49, ***p*** = 0.000481
**Item 10:** If someone with a health problem has genome sequencing, they will always find helpful information about the cause of the problem (false)	71 (24.6%)	98 (33.9%)	*Z* = −3.17, ***p*** = 0.002	61 (23.0%)	143 (54.0%)	*Z* = −7.49, ***p*** = 6.97^**−14**^

Note: *p* values in bold indicate significance over 0.05

### Change in objective knowledge in sample overall

**Fig. 1:**
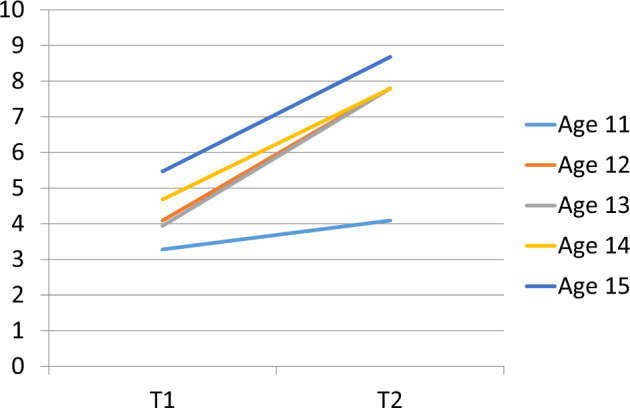
Change score by age in sample overall.

Objective knowledge increased from T1 to T2 in the sample overall, i.e. after combining the data from Studies 1 and 2, (mean 4.24 vs 7.6, *t* = 32.16, *p* < 0.001). It also increased for both girls and boys, for all ages (Fig. 1), and at both primary and secondary schools (*p* < 0.001 for all).

### Associations between objective knowledge and participant characteristics

#### At baseline

As one would expect, there was a positive correlation between age and knowledge with older children having greater knowledge than younger children (*p* < 0.001). Boys had a mean higher knowledge score at T1 than girls (mean 4.44 vs 4.09 respectively, *F* = 1.66, *p* < 0.001). Secondary schools also had a higher mean score than primary schools (4.43 vs 2.54, *F* = 6.88, *p* < 0.001). All of the variables (age, gender, school type) remained associated with T1 knowledge when entered into a multivariable linear regression.

#### After watching the animation

T2 knowledge (after watching the animation) was associated with being older (*p* < 0.001) and was higher among secondary schools than primary schools (mean 7.93 vs 4.71 respectively, *F* = 2.48, *P* < 0.001). There was no longer a statistically significant association with gender (mean 7.63 vs 7.59 respectively, *F* = 1.68, *p* = 0.20). In multivariable analysis, both age and school type remained associated with knowledge.

#### Change score

The increase in knowledge (T1–T2 change score) was greater for girls than it was for boys (mean change score 3.50 vs 3.19 respectively, *F* = 12.69, *p* < 0.001), and for secondary schools compared with primary schools (3.50 vs 2.18, *p* < 0.001). There was also a difference between age groups but the association was not linear. The greatest increase was for 13 year olds (mean change score 3.84), the lowest was 11 year olds (mean change score 2.68). In multivariable analysis, the only association that remained statistically significant was school type (secondary vs primary).

### Attitudes

After watching the animations, the majority of participants in both Study 1 and Study 2 felt they understood the benefits of genome sequencing (75.4% and 76.6%), thought genome sequencing was a good thing (74.7% and 70.6% respectively),and thought genome sequencing was helpful (81.3% and 83.0% respectively) and interesting (73.5% and 80.4% respectively) (Table 4). In Study 1, only 17.3% of participants felt they understood the limitations of genome sequencing after watching the animation. However, this was higher in Study 2, with 58.5% of participants indicating they understood the limitations of GS (*X*^2^(2) = 138.70, *p* < 0.001) even after controlling for gender and age (OR 10.26, 95% CI 6.46–16.30, *p* < 0.001). There were no other statistically significant attitudinal differences towards genome sequencing between Study 1 and Study 2.

**Table 4:** Attitude towards genome sequencing and satisfaction with information comparing Study 1 and Study 2.

Construct/measure	Study 1	Study 2	Sig.
Attitude			
**Would you want to have genome sequencing?**			
Yes	133 (46.0%)	131 (49.4%)	*X*^2^(3) = 10.10, ***p*** = 0.018
No	0 (0.0%)	4 (1.5%)	
Not sure	26 (9.0%)	35 (13.2%)	
I would want more information before making a decision	123 (42.6%)	88 (33.2%)	
**I feel I understand the benefits of genome sequencing**			
Agree	218 (75.4%)	203 (76.6%)	*X*^2^(2) = 3.12, *p* = 0.21
Disagree	8 (2.8%)	2 (0.8%)	
Not sure	61 (21.1%)	55 (20.8%)	
**I feel I understand the limitations of genome sequencing**			
Agree	50 (17.3%)	155 (58.5%)	*X*^2^(2) = 138.70, ***p*** = 7.62^**−31**^
Disagree	141 (48.8%)	23 (8.7%)	
Not sure	97 (33.6%)	84 (31.7%)	
**I feel the decision to have/not have genome sequencing would be easy for me to make**			
Agree	87 (30.1%)	109 (41.1%)	*X*^2^(2) = 11.56, ***p*** = 0.003
Disagree	71 (24.6%)	39 (14.7%)	
Not sure	129 (44.6%)	115 (43.4%)	
**Genome sequencing is:**			
A bad thing	3 (1.0%)	4 (1.5%)	*X*^2^(2) = 1.15, *p* = 0.56
A good thing	216 (74.7%)	187 (70.6%)	
Neither	66 (22.8%)	69 (26.0%)	
**Genome sequencing is:**			
Harmful	3 (1.0%)	6 (2.3%)	*X*^2^(2) = 1.60, *p* = 0.45
Helpful	235 (81.3%)	220 (83.0%)	
Neither	42 (14.5%)	34 (12.8%)	
**Genome sequencing is:**			
Boring	11 (3.8%)	10 (3.8%)	*X*^2^(2) = 4.24, *p* = 0.12
Interesting	213 (73.5%)	213 (80.4%)	
Neither	62 (21.5%)	39 (14.7%)	
**Was the animation easy or hard to understand?**			
Very/quite easy	278 (96.2%)	242 (91.3%)	*X*^2^(1) = 4.61, ***p*** = 0.032
Very/quite hard	10 (3.5%)	20 (7.5%)	
**The amount of information in the animation was:**			
Too much	10 (3.5%)	19 (7.2%)	*X*^2^(2) = 5.82, *p* = 0.054
Too little	24 (8.3%)	30 (11.3%)	
The right amount	255 (88.2%)	214 (80.8%)	
**The length of the animation was:**			
Too long	6 (2.1%)	19 (7.2%)	*X*^2^(2) = 10.48, ***p*** = 0.005
Too short	46 (15.9%)	29 (10.9%)	
The right amount	237 (82.0%)	216 (81.5%)	
**What did you think about the way the animation looked?**			
I liked it very much	115 (39.8%)	88 (33.2%)	*X*^2^(2) = 3.02, *p* = 0.221
I quite liked it	163 (56.4%)	168 (63.4%)	
I didn’t like it	11 (3.8%)	8 (3.0%)	
**Did you learn anything new?**			
Yes	274 (94.8%)	227 (85.7%)	*X*^2^(2) = 12.64, ***p*** = 0.002^**a**^
No	4 (1.4%)	9 (3.4%)	
Not sure	11 (3.8%)	28 (10.6%)	
**Would you have found this animation helpful if you were making a decision about having genome sequencing?**			
Yes	208 (72.0%)	196 (74.0%)	*X*^2^(2) = 1.00, *p* = 0.606
No	12 (4.2%)	13 (4.9)	
Don’t know	69 (23.9%)	54 (20.4%)	

^a^After controlling for age and gender: OR −0.46, 95% CI 0.23–0.94, *p* = 0.033

Note: *p* values in bold indicate significance over 0.05

### Satisfaction

There was high overall satisfaction with the animation (Table 4). The majority of pupils thought the animation was very or quite easy to understand (96.2% Study 1 and 91.3% Study 2), had the right amount of information (88.2% Study 1 and 80.8% Study 2) was the right length (82.0% Study 1 and 81.5% Study 2), very much liked or liked the way the animation looked (96.2% Study 1 and 96.6% Study 2), learnt something new (94.8% Study 1 and 85.7% Study 2) and would find the animation helpful if making a decision about GS (72.0% Study 1 and 74.0% Study 2). There were no statistically significant differences between the two studies for any of the satisfaction questions.

### Qualitative evaluation

Ten families who watched the animation as part of a consent appointment for the 100,000 Genomes Project were invited to take part in an in-depth qualitative interview. One declined as they were in a hurry and nine families took part (9 probands, 3 siblings and 14 parents). The three consenters who had been showing the animation during 100,000 Genomes Project appointments also took part in an interview. Key themes along with example quotes can be found in Supplementary Material 6.

#### Theme 1: The animation was an effective way of enhancing understanding about genome sequencing

Young people found the animation to be a “fun”, “easy to understand” and engaging way of learning about GS.

“It made it better because I actually knew what they were talking about”….I’d have been thinking they were talking on another planet!” – P8 female age 11

Parents also felt they better understood the concept of GS after watching the animation. Consenters commented that the animation was useful in helping them “gauge what [patients and their families] understood”. It also prompted participants to ask questions and opened up a dialogue between the consenter and the family.

“When I showed them this movie, after the movie they said ‘oh yes, I have this question and this question and at this moment how long would it take for a result’ or things like that’” – C1

One consenter commented that it would be useful to show the second animation to explain why they might not get a result from GS.

#### Theme 2: The animation helped young people feel more comfortable and engaged in the (research) process

Watching the animation prepared young people for what would happen during the appointment, with young people being “less nervous” about the blood test because they knew why it was being done.

“Because, like, instead of, like, being nervous about the blood test and stuff, you could have it before so we could be less nervous instead…. Because we know why we’re taking the blood” – P7 male age 11

Parents also felt that because their child was “more at ease” with the process, it made them “feel a bit more at ease about it”. Young people spoke about feeling more “positive” about GS after watching the animation because it helped them understand that “you might get a result” and it could “help other people”. The animation also led to young people being more curious around their future, for example, whether certain genes would continue to be passed down in their family. Consenters found that an outcome of watching the animation was that it empowered the young people in the appointments to take a more active role in the decision-making process about GS; “they were definitely more engaged and definitely more part of the consent process”. It also facilitated engagement between the consenter and the young person as opposed to just the parents:

“I found it quite a useful way to kind of getting engagement with the kids, because sometimes the consent discussion can be quite adult orientated and it ends up mostly being a discussion between me and the parents without so much involvement from children” – C3

#### Theme 3: Showing the animation at the start of the appointment was most effective

Watching the animation at the start of the appointment, rather than later in the appointment, was perceived by both parents and young people to be the most effective time to show it as it prepared young people for “what’s going to happen” and allowed time for the consenter to answer any questions that were raised.

“I think [the beginning] is a good time because you can watch it and you can get told about it again if you don’t understand anything.” – P9 girl age 11

In addition, consenters preferred to show the animation at the beginning:

“Mostly I chose to show it at the beginning of the sessions and I thought it was a nice icebreaker because it was something a little bit more informal than going into regulation and rights, etc.” – C2

## Discussion

Our results show that the animations objectively and subjectively improved young people’s understanding of what a genome is and what genome sequencing is, and the addition of a second animation significantly improved young people’s understanding of the limitations and uncertainties of this technology. The animations were well received, with the majority of participants scoring the animations highly on understandability, content, length and look. The first animation was also found to help ease concerns about the testing process, create enthusiasm about the potential benefits to them and others, and empower young people to take a more active role in the decision-making process. As GS moves into clinical practice, the ‘My Genome Sequencing’ animations fill an important gap by providing an educational resource which has been designed with young people and is effective in improving knowledge and understanding. The next step will be to formally evaluate the impact of the animations in a clinical setting.

Our study adds to the growing body of literature showing that animations are an effective way of increasing genomic literacy amongst young people [3, 13]. A recent, small study in the USA by Sabatello et al. [13] found that amongst 43 adolescents (aged 14–17 years), both objective and subjective knowledge about genome sequencing increased after watching an animation designed for adults. Our study builds on the work by Sabatello et al. because of our larger sample size, the inclusion of qualitative findings, and because the animation used in that study was not specifically developed for or co-designed with young people.

The addition of the second animation was found to improve young people’s understanding of the limitations and uncertainties around GS, namely that they may not get any meaningful information from having GS. Promoting a realistic understanding of the benefits and limitations has been identified as important [17, 24] particularly given that some patients may have unrealistic expectations of genomic technology [25] or may be disappointed with the scope of the results returned [26]. Currently, sequencing may only successfully identify a genetic cause in around 40% of previously unsolved paediatric cases [27]. However, there is the possibility that as genomic knowledge improves a cause could be identified with re-analysis in the future [28]. A critical component of obtaining consent for GS will be ensuring patients are prepared for the limitations and uncertainties around GS, and that expectations are managed [29].

### Strengths and limitations

A key strength of this study is the iterative co-design process used to develop the animations in which we tested out the script and animations at varying stages of development with patients and school pupils as well as with an advisory team which comprised experts from a range of backgrounds. The use of more social and user-centred processes in the design of health interventions involving relevant stakeholders as co-designers, is being increasingly recognised [30, 31]. We employed a range of research methods in the development of the animations including a review of the currently available online resources as well as qualitative interviews with young people and ‘think-aloud’ cognitive interviews to test the script and storyboard. The importance of using a range of research methods to generate the evidence based on which to develop health interventions has been acknowledged as good practice in ensuring utility and acceptability [32]. Our study also adds to the growing body of literature on co-design techniques for educational resources [33–35]. We also employed a mixed-methods design to evaluate the animation which provided a richer and more nuanced picture of how the animations were received.

A limitation of our study is that only participants that could understand English were included in the evaluation. This could potentially have excluded some young people/parents from the study. Since our animations were developed, we have translated them into Turkish and Bengali (two of the languages spoken most frequently by patients/parents of children with rare diseases at Great Ormond Street Hospital) to increase their accessibility. The translations have been added as subtitles rather than as a voice-over (due to cost), although we acknowledge that voice-over would have been preferable to enhance understanding and facilitate deep-learning. Future research could look at the views of young people where English is not their first language. A further limitation is that the quantitative evaluation was only conducted with school pupils and not patients with rare diseases. Young people with rare diseases may have prior knowledge about genome sequencing or may have different information needs. Therefore, the animations may be less effective in this group. Further research is therefore required. Finally, none of the participants that contributed to the development and evaluation of the animation had declined to take part in the 100,000 Genomes Project. Decliners may have different views towards what information should be presented in information resources about genome sequencing.

In conclusion, we have developed an information resource about GS which has been co-designed with young people, is freely available online, which has been shown to improve knowledge, and was well received by patients, families and health professionals. The first animation was shown to be effective at improving knowledge around what a genome is, genomic variation and genome sequencing; the second animation improved knowledge of the limitations of genome sequencing. We therefore recommend that young people watch *both* animations in order to have a broader understanding of the potential outcomes of genome sequencing. Further research to compare our animations with a pamphlet (with the same script) is ongoing to assess whether an animation is more effective in terms of improving knowledge than written information alone. Future research could focus on formally evaluating the animations in a clinical setting and testing alternate ways of showing them e.g. during the appointment itself, whilst waiting for the appointment, and/or at the results disclosure appointment.

## Supplementary information

Supplementary Material 1_Development of the animation

Supplementary Material 2_Review of online existing information sources

Supplementary Material 3_Questions about whole genome sequencing

Supplementary Material 4_Animated video topics

Supplementary Material 5_Questionnaire

Supplementary Material 6_Qualitative themes and supporting quotes
