# Supplementary material for: Development and mixed-methods evaluation of an online animation for young people about genome sequencing
Source: Eur J Hum Genet. 2020 Jan 2;28(7):896–906. doi: 10.1038/s41431-019-0564-5 (PMC7316978; doi:10.1038/s41431-019-0564-5)
Supplement: Supplementary file 1 — Supplementary Material 1_Development of the animation [file 41431_2019_564_MOESM1_ESM.docx]

# Development of the animation

The animation was co-designed with 1) young people taking part in the 100,000 Genomes Project, 2) school pupils,3) members of a children’s hospital Young Person’s Advisory Group, and 4) members of an expert working group which consisted of two science teachers, a geneticist, two genetic counsellors, a clinical scientist, two behavioural scientists with expertise in genetics, a commercial digital communications expert and two patient advocates from the support groups Genetic Alliance UK and Unique: The Rare Chromosome Support Group. The development consisted of three key phases (Figure 1).

**Figure 1: Phases of animation development**


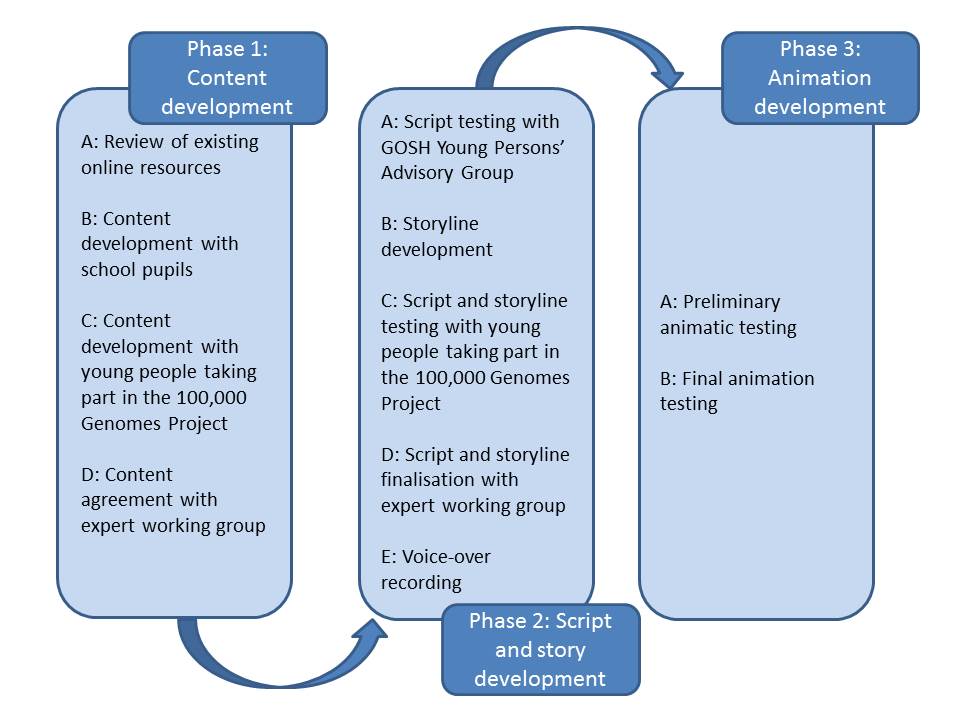


# Phase 1: Content development

### A: Review of existing online resources: A review of existing online educational websites about genomics and WGS was conducted in December 2015. (see: Supplementary Material and Methods 2). Twenty-three online resources (including videos, animations, infographics) from fifteen online platforms were identified. Only one appeared to be aimed specifically at young people (Genetics 101 developed by 23andMe and Khan Academy) but it did not provide information about WGS. We drew on these websites when developing a list of potential topics to include in the animation.

B: Content development with school pupils: In June 2016 CL, SS and/or LC visited two schools (one primary school and one secondary school) and spoke with pupils in five classes aged between 11-15 years. During each class visit the pupils were shown part of an animation about WGS developed by Sanderson et al. (“Whole Genome Sequencing and You”)^1^. Feedback on the animation was then sought: we asked pupils what aspects of the animation they found easy to understand and what aspects they found difficult to understand and what aspects of the animation they liked and what they disliked. The feedback included that pupils liked the idea of zooming into the body to see the cells, chromosomes and DNA and they liked seeing the thousands of As, Cs, Gs and Ts on the screen.

We then presented pupils with three analogies to explain what a genome is, which had been suggested by a genetic counsellor in our expert working group, and asked them which analogy they liked best. Analogies have been shown to promote comprehension and memory of scientific concepts.^2^ The analogies were: 1) explaining a genome as being like a recipe to bake a cake, 2) explaining a genome as being like an instruction manual for building blocks, and 3) explaining a genome as being like the code for a computer game. This third analogy was the most popular and appealed to both boys and girls. Finally, we asked pupils what information they thought was important to present in an animation about WGS, and what questions they would want answered if they were considering having WGS. Responses included understanding what our genome does, what genome sequencing involves and whether there were any risks from genome sequencing (see Supplementary Material and Methods 3).

C: Content development with young people taking part in the 100,000 Genomes Project: Between January and June 2017, we conducted four semi-structured telephone interviews with young people aged 11-15 taking part in the 100,000 Genomes Project to explore their experience of WGS. During those interviews we asked participants what information they thought should be included in an animation about WGS. Responses included explaining how our genes can affect our health, that it is done via a blood test, and explaining what scientists do with the blood once it gets to the laboratory (see Supplementary Material and Methods 3).

D: Content agreement with expert working group: A topics grid informed through Phases 1A, 1B and 1C was developed and the expert group was asked to prioritise which topics they thought should be included in the animation. The topics grid was grouped into four key domains: 1.genetics – the basics, 2. what is whole genome sequencing, 3. what results might you receive and 4. making your decision (benefits, risks, limitations, uncertainties) (see Supplementary Material and Methods 4).

E: First draft of script developed: Based on the feedback from phase 1D, a draft script was developed by CL and SS in September 2016. In order not to overload viewers with too much information (limited capacity assumption) the script focused on two topic domains, - ‘genetics – the basics’ and ‘what is a genome sequence’. In addition, the aim was to develop a script that was around 300-350 words in order that the total length of the animation would be no longer than three minutes. Videos up to three minutes in length have been shown to have the highest engagement with students.^3^ The draft script was revised multiple times with input from the animation company. At this stage it was decided that instead of describing the genome as being like the code for a computer game, we would use the idea of the genome as being like the code that gives robots instructions as it was felt that this would be more visually engaging. The draft script was then sent to a professional script editor who made suggestions to tighten the script without affecting the meaning.

## **Phase 2: Script and story development**

A: Script testing with Young Persons’ Advisory Group: The draft script was tested out with the Great Ormond Street Hospital Young Persons’ Advisory Group (GOSH YPAG). The GOSH YPAG is a group of around 10-15 young people (aged between 8 and 21 years), who are current or previous patients at GOSH and who provide feedback on research that is relevant to children and young people. A ‘think-aloud’ method was used whereby CL read the entire script out aloud initially, and then went back and read the script aloud one sentence at a time. After each sentence, the group was asked to comment on whether the sentence was clear, and comment on any word or phrase that was difficult to understand and suggest alternative wording. In some cases, multiple options for a word or sentence were presented and the group was asked to comment on which they preferred. For example, we asked the group whether they preferred the word ‘change’ or ‘glitch’ to describe a variant in a gene. The word ‘change’ is used in the 11-15 year old Participant Information Sheet developed by the 100,000 Genomes Project.^4^ The word ‘glitch’ is used in the animation ‘Socialising the Genome’.^5^. Most YPAG participants preferred the word ‘glitch’ as they thought it would fit in better with the robot analogy and was a more ‘engaging’ word for young people.

B: Visual story development: For each line of the script, a visual accompaniment or ‘story’ was produced by the animation company to highlight what would be happening in the animation. The story would revolve around a young girl and her family who have whole genome sequencing. The robots would be used to highlight how our genome affects our health, including what happens if there is a ‘glitch’ in the code (in the animation, one of the robot’s wheels falls off). In this way we could be more playful when highlighting the affect the genome has on the body.

C: Script and story testing with young people taking part in the 100,000 Genomes Project: The four young people who had taken part in the interviews (Phase 1C) were invited to comment on the revised script and story. This was done via a telephone interview. The four young people gave feedback on the draft script (using the ‘think-aloud’ technique described above in Phase 2A) and provided feedback on the proposed story, in particular whether they liked the idea of the robot and whether they thought that story would be engaging. A few minor changes to the storyline and script were made at this stage. For example, in the original storyline, in one scene the doctor shows the family the results from genome sequencing on a piece of paper. It was suggested that instead the doctor could show the family the results on a digital tablet.

D: Script and storyline finalisation with expert working group: The revised script and story were sent to the expert working group for feedback to ensure there was nothing in the script or story that was scientifically inaccurate or might be inappropriate or upsetting for young people. Some minor changes to the script were made. A final version of the script was approved by the expert working group.

## E: Voice-over recording: Two interviewees from Phase 2C were invited to potentially provide the voice-over for the animation. Following parental permission, the interviewees audio-recorded themselves reading the script. After listening to the two audio-recordings, CL and a member of the animation company selected a narrator (female, aged 12). The voice-over was then professionally recorded at a recording studio in Soho, London in October 2016.

## **Phase 3: Animation development**

A: Preliminary animatic testing: Once the voice-over had been recorded, an ‘animatic’ (a preliminary version of the animation consisting of a successive series of animated storyboards) was developed by the animation company, which was timed with the voice-over. In November 2016, we tested out the animatic with pupils aged 11-15 at a secondary school in London. We asked pupils specifically to provide feedback as they were watching the animatic on whether there were any visuals that were unclear or did not match what was being narrated. The pupils suggested some minor change to the animatic, for example, they suggested that to highlight how long it was taking for the main character to read her entire genome sequence, we could make her hair go grey. Pupils were also presented with still shots from four different animators, all of whom had designed animations aimed at young people but whom had very different animation styles. Pupils were asked to select their preferred animator. Finally, pupils were asked to listen to three different music tracks and select which one they thought would work best with the animation.

B: Final animation testing: Following feedback from pupils in Phase 3A, an animator was selected who then developed the animation. In February 2017 a draft of the animation was tested out with the GOSH YPAG and the four young people from Phase 2B. A couple of minor changes were made at this stage, for example, reducing the size of a needle on the syringe when the girl is having a blood test, and making it clearer that the robot’s wheel is missing by highlighting it in red. The animation was then viewed and approved by the expert working group. The 2.5 minute animation ‘My Genome Sequence’ was uploaded onto the GOSH YouTube channel in March 2017 (<http://bit.ly/mygenomesequence>) and subsequently English subtitles were added to enhance accessibility.

## **Development of second animation; “My Genome Sequence part 2”**

Between October 2017 and July 2018 the second animation ‘My Genome Sequence Part 2’ was developed to address the two domains, ‘what results might you receive (including secondary findings)’ and ‘making your decision (benefits, risks, limitations, uncertainties)’ which were not covered in-depth in the first animation. The recent AGMC statement on engaging children and adolescents in exome/genome sequencing conversations underscored the importance of ensuring young people understand that sequencing “may not find the answer related to the indication for testing” and that “the test might uncover unexpected information unrelated to the indication for the testing”.^6^ The development of the follow-up 2.5 minute animation followed the same developmental process as the first animation (Phase 3 to Phase 6) whereby the script, storyline and animatic underwent testing with the GOSH YPAG, pupils from two schools in London and four young people taking part in the 100,000 Genomes Project (the schools and young people were different from those who took part in the development of the first animation). The follow-up animation was uploaded onto the GOSH YouTube channel in August 2018 (<http://bit.ly/mygenomesequence2>).

1. Sanderson SC, Suckiel SA, Zweig M, Bottinger EP, Jabs EW, Richardson LD. Development and preliminary evaluation of an online educational video about whole-genome sequencing for research participants, patients, and the general public. Genetics in medicine : official journal of the American College of Medical Genetics*.* 2015;18(5):501-512.

2. Halpern DF, Hansen C, Riefer D. Analogies as an aid to understanding and memory. Journal of Educational Psychology*.* 1990;82:298-305.

3. Guo PJ, Kim J, Rubin R. How video production affects student engagement: an empirical study of MOOC videos. Proceedings of the first ACM conference on Learning @ scale conference*.* 2014:41-50.

4. Genomics England, <https://www.genomicsengland.co.uk/taking-part/patient-information-sheets-and-consent-forms/>. Accessed Accessed July 23, 2018. .

5. Anna Middleton, 2016. <https://genetube.org/6fd741?fv=#/start-video>.

6. Bush LW, Bartoshesky LE, David KL, Wilfond B, Williams JL, Holm IA. Pediatric clinical exome/genome sequencing and the engagement process: encouraging active conversation with the older child and adolescent: points to consider-a statement of the American College of Medical Genetics and Genomics (ACMG). Genetics in medicine : official journal of the American College of Medical Genetics*.* 2018.
