# Supplementary material for: Development and mixed-methods evaluation of an online animation for young people about genome sequencing
Source: Eur J Hum Genet. 2020 Jan 2;28(7):896–906. doi: 10.1038/s41431-019-0564-5 (PMC7316978; doi:10.1038/s41431-019-0564-5)
Supplement: Supplementary file 2 — Supplementary Material 2_Review of online existing information sources [file 41431_2019_564_MOESM2_ESM.docx]

Review of online existing information sources 3.12.15

**Search strategy**

Search engines: Google, Bing & You Tube

Search terms: ‘whole genome sequencing’, ‘genome sequencing’ ‘genomics’

| Produced by | URL | Information delivery | What is covered | Strengths and Limitations |
| --- | --- | --- | --- | --- |
| National Genetics and Genomics Education Centre | <http://www.geneticseducation.nhs.uk/>  <http://www.geneticseducation.nhs.uk/mededu/genes-to-genome>  http://www.geneticseducation.nhs.uk/genomic-healthcare  http://www.geneticseducation.nhs.uk/for-healthcare-educators/clinical-images  <https://vimeo.com/78906181>  <https://www.flickr.com/photos/119980645%40N06/?rb=1>  (free image library) | Online text  Clinical images, animations and videos  Information divided into the following sections: I’M LEARNING I’M TEACHING  I’M IN CLINICAL PRACTICE  GENOMIC HEALTHCARE  RESOURCES  Video animation series: Introduction to genomics | Whole section on genetics (genes to genome): DNA, genes, chromosomes, genetic code, inheritance patternsSection on genomics in healthcare:Translational genomics: the path from genomic insight to clinical applicationsExamples of genomic healthcareRefining diagnosisIndividualising clinical carePredicting drug effectsDeveloping new therapies Video of Leslie Biesecker discussing whole genome sequencing  Clinical images, animations and videos Animated videos on inheritance patterns,[Using genomic information to predict the effect of drugs](http://vimeo.com/83843207)[Using genomic information to predict and prevent adverse drug reactions](http://vimeo.com/83845471) How can your genome affect your health? | Quite high level –it is aimed at healthcare professionals rather than patients.  A lot of information to go through. Not focused on patient perspective |
| NHS England Genomics Education Programme | <https://www.genomicseducation.hee.nhs.uk/resources/videos> | Video – Introducing genomics in healthcare  Video – Rare disease, a families journey | Intro to genetics/genomics  How it is being used  Stratified/personalised medicine  Diabetes/cancer  Genome sequencing of MRSA  Molecular ‘lab in a box’  100,000 Genomes Project  Health Education England  Discussed 1 particular rare disease patient and how 100,000 Genomes Project is helping | Aimed at healthcare professionals  Talks about 100,000 Genomes project  Talks about 100,000 Genomes Project |
| Genomics England | <http://www.genomicsengland.co.uk/taking-part/participant-stories/>  <http://www.genomicsengland.co.uk/the-100000-genomes-project/understanding-genomics/> | Video – participant stories  Understanding genomics – infographic, animated infographic and video | Stories from patients who have taken part in the 100,000 Genomes Project  What is a genome? (infographic)  How do you sequence a genome? (infographic)  Genomics in healthcare (video – same as above)  What happens to the data in the project? (animated infographic) | Aimed at patients |
| 23 and Me and Khan academy -  Genetics 101 | <https://www.youtube.com/watch?v=ubq4eu_TDFc&list=PLF9969C74FAAD2BF9> | Animation | What are genes?  What are SNPs?  What are phenotypes?  Why not Y? | Had over half a million views. Appears to be aimed at young people. Uses animated images. 810/855 ‘likes’  Doesn’t specifically address genome sequencing |
| Genes and health | <http://www.genesandhealth.org/syl/node/63> | Online information with images & animation.  2 videos | Genes and your health – genes made easy  Easy explanation of genes and science  Genes, chromosomes, where do your genes come from, how do genes affect your health, genetic conditions, personalised medicine  Watch the YourGenome from DNA to protein video to see how it all works  Zoom in on your genome | Explains basic genetic concepts but not much information on genomics. |
| Your Genome – Wellcome Trust Sanger Institute | <http://www.yourgenome.org/facts/what-is-a-genome>  <http://www.yourgenome.org/videos/> | What is a genome? Webpage  ‘Zoom in on your genome’ animated video | Explanation of: Gene, genome, DNA, chromosome, inheritance  An array of short films giving insight into what is happening in genomics and how it is helping in the fight against human disease. Animations show how we sequence DNA and other techniques. Includes “my career in genomics”, “the human genome project”, “DNA sequencing”, | Clear and concise explanations  Video ‘zoom in on your genome’ provides a good short explanation  Some of the videos quite complex. Some of the videos aimed at graduates  Good summary of human genome project  Some of the animations e.g. ‘how the human genome was sequenced’ do not have audio – only wording |
| Sanger survey on genomethics | <https://survey.sanger.ac.uk/genomethics/> | Survey with embedded videos | Sharing of Pertinent Findings  [Sharing of Incidental Findings](https://survey.sanger.ac.uk/genomethics/)  [Categorizing Incidental Findings](https://survey.sanger.ac.uk/genomethics/)  [Relations with Risk](https://survey.sanger.ac.uk/genomethics/)  [Raw data](https://survey.sanger.ac.uk/genomethics/)  [Duty of Genomic Researchers](https://survey.sanger.ac.uk/genomethics/)  [Filter of Genomic Information](https://survey.sanger.ac.uk/genomethics/)  [Consent for genomic research](https://survey.sanger.ac.uk/genomethics/) | Focused mainly on some of the ethical issues associated with genomic research studies and incidental findings.  Doesn’t include background information about genomics |
| Icahn School of Medicine | <https://www.youtube.com/watch?v=IXamRS85hXU&hd=1> | “Whole genome sequencing and you” video | Genetics: the basics  What is whole genome sequencing – physical traits, ancestry, risk of common diseases and psychiatric disorders, genetic disorders, carrier status, VUS, pharmacogenetics  Making your decision – benefits, risks and limitations, privacy | Has undergone formal review process with adults (18-89 years) and was well received |
| National Human Genome institute | <http://www.genome.gov/18016863> | Fact sheet – A brief guide to genetics | DNA, Genes and Genomics  DNA sequencing  The Human Genome Project  Implications of Genomics for Medical Science | Doesn’t address whole genome sequencing |
| PHG Foundation | <http://www.phgfoundation.org/file/10365/> | 4 page summary leaflet of main report | Whole genome sequencing – clinical impact and implications for health services | Aimed at health professionals |
| Learn Genetics Utah | <http://learn.genetics.utah.edu> | online animated videos | Tour of basic genetics  Human health  Cell biology | Doesn’t specifically address whole genome sequencing |
| Genetics Home Reference | <http://ghr.nlm.nih.gov/handbook.pdf> | Help me understand genetics handbook – 203 pages. | 1 chapter on genomic research  SNPs  GWA studies  HapMap project  ENCODE project  Pharmacogenetics  Advances in DNA sequencing | Very dense  Does not provide a summary  Text based |
| Illumina website – Genetics 101 | [www.everygenome.com](http://www.everygenome.com)  <http://www.illumina.com/clinical/illumina_clinical_laboratory/genomics-101.html> | Video  Webpage – Genomics 101 | Case study of a patient with a rare disease who benefited from WGS.  Page itself aimed at clinicians.  Reading the genome  DNA as genomic encyclopaedia  Genomes and inherited disease  DNA changes | Video gives a good example of how WGS can help patients, but doesn’t give much information about WGS itself and what it is.  The genomics 101 page is patient friendly |
| Genes in Life | <http://www.genesinlife.org/testing-services/testing-genetic-conditions/whole-genome-sequencing> | What is whole genome sequencing? Webpage | What is whole genome sequencing?  How can I be tested?  What do the test results mean?  Questions to consider  Also has a Genetics 101 and Genes and Your Health section on the website | Seems to be written for patients as language is very clear and simple. |
| How to sequence the human genome | <https://www.youtube.com/watch?v=MvuYATh7Y74> | Ted Ed |  | Relatively complex  some useful analogies |
