# Supplementary material for: Development and mixed-methods evaluation of an online animation for young people about genome sequencing
Source: Eur J Hum Genet. 2020 Jan 2;28(7):896–906. doi: 10.1038/s41431-019-0564-5 (PMC7316978; doi:10.1038/s41431-019-0564-5)
Supplement: Supplementary file 3 — Supplementary Material 3_Questions about whole genome sequencing [file 41431_2019_564_MOESM3_ESM.docx]

# Questions identified by school pupils & young people in the 100,000 Genomes Project about whole genome sequencing

# Genetics - the basics

- What is DNA?
- What is a gene?
- What is a genome?
- What does our genome do?
- Do our genomes all look the same? Are they the same as our parents?
- Why does our genome affect our health?

**What is whole genome sequencing**

- What is whole genome sequencing?
- How do you do whole genome sequencing? What is the process?
- Will you always get an answer from genome sequencing?
- How accurate are the results?

**Questions around the process of genome sequencing**

- What do they do with the blood? Who does the test?
- How long will it take to do the test? How long will it take to get the results back? How will I get the results back?
- How many times do you do the test to make sure it’s right?
- Do I have to have the test?

**What results you might receive**

- What could be the possible result? What information might I get back?
- Can you get results for other things in addition to the main genetic conditions?
- What if you find something? What are the consequences if I find out I have a genetic condition?
- What happens after I get the result? What is the treatment?

# Benefits

- What are the benefits?
- In what ways will it benefit me? Will it benefit other people?

# Risks, limitations, uncertainties

- What are the risks? What are the disadvantages?
- Will you always get an answer from genome sequencing?
- Is it safe?
- How many people have had it done?
- Will it work for everyone, because everyone’s genes are different?
- Can the information be used against me in any way?
