# Supplementary material for: Development and mixed-methods evaluation of an online animation for young people about genome sequencing
Source: Eur J Hum Genet. 2020 Jan 2;28(7):896–906. doi: 10.1038/s41431-019-0564-5 (PMC7316978; doi:10.1038/s41431-019-0564-5)
Supplement: Supplementary file 4 — Supplementary Material 4_Animated video topics [file 41431_2019_564_MOESM4_ESM.docx]

| Section | **Animated video**  **topics** |
| --- | --- |
| **1. Genetics: the basics** | - Bodies made of millions of cells which contain DNA |
|  | - Our DNA act like set of instructions and control how our body works |
|  | - A genome is a person’s complete set of DNA |
|  | - Genome made up of 6 billion chemical ‘letters’ AGTC |
|  | - Order of DNA is your genome sequence |
|  | - Each person’s genome sequence is unique |
|  | - Variation in the DNA sequence can cause body not to function properly and may cause a genetic condition |
|  | - Genes are short sections of DNA |
|  | - Inherit our genes from our parents |
|  | - Humans have about 20,000 genes |
|  | - Genes code for proteins which do most of work in body |
|  | - Genes packaged on chromosomes |
| **2. What is whole genome sequencing?** | - Procedures |
|  | - Genome sequencing is a technology that involves sequencing or writing down exact order of all the As Cs Gs and Ts |
|  | - Other genetic tests only look at certain sections of the DNA in your genome, WGS looks at almost all of it |
|  | - Blood test or saliva sample |
|  | - Amount of blood will depend how old you are but varies between 6-12ml (1-2 teaspoons) |
|  | - Blood sent to laboratory where DNA is extracted using chemicals |
|  | - Use very high powered machines to read the DNA sequence |
|  | - Interpret sequence by comparing it to a reference sequence |
|  | - Other close relatives (usually parents) may also be tested for comparative purposes |
|  | - Timeframe for getting results |
|  | - How you will receive the results |
|  | - Test accuracy |
| **3. What results you may receive?** | - Identify genetic cause of you/your child’s condition |
|  | - - Additional findings about risk of disease such as cancer or other rare genetic conditions |
|  | - - Carrier status |
|  | - - Variants of unknown significance |
|  | - - Your likely response to therapeutic drugs |
|  | - - You can choose whether you want to know about these additional findings |
| **4. Making your decision** | - Benefits |
|  | - - Getting a diagnosis. Find out ‘cause’ for you/your child’s condition |
|  | - - If you get a result might help identify treatment/monitoring/prevention |
|  | - - Reproductive decision-making |
|  | - - Alert other family members of potential health risks |
|  | - - Psychological/social benefits of diagnosis e.g. end of ‘diagnostic odyssey’, connecting with people with same condition |
|  | - - Contribute to genomics research and improve health of future generations |
|  | - Risks |
|  | - - Might not always find a genetic cause for condition – can be disappointing |
|  | - - Blood test so might feel slight sting and leave some bruising |
|  | - - Worry if find out something unexpected |
|  | - - Potential loss of privacy |
|  | - - Concerns around insurance |
|  | - Limitations |
|  | - - Interpretation of DNA sequence may not provide any information about your condition |
|  | - - Technology is still in its infancy so much we still can’t interpret |
|  | - Privacy |
|  | - - Genome sequencing is voluntary |
|  | - - Results are confidential |
