# Supplementary material for: Development and mixed-methods evaluation of an online animation for young people about genome sequencing
Source: Eur J Hum Genet. 2020 Jan 2;28(7):896–906. doi: 10.1038/s41431-019-0564-5 (PMC7316978; doi:10.1038/s41431-019-0564-5)
Supplement: Supplementary file 5 — Supplementary Material 5_Questionnaire [file 41431_2019_564_MOESM5_ESM.pdf]

QUESTIONNAIRE 1

Great Ormond Street Hospital for Children NHS
NHS Foundation Trust

Some questions about you

1. Are you a:

☐ Boy

☐ Girl

2. How old are you?

☐

Some questions about your understanding of genetics

3. How would you describe your understanding of genetics?

☐ None

☐ Some

☐ Good

4. Have you heard of these words before?

DNA	Yes ☐	No ☐
Gene	Yes ☐	No ☐
Chromosome	Yes ☐	No ☐
Genome	Yes ☐	No ☐
Genome sequencing	Yes ☐	No ☐

5. Do you know what these words mean?

DNA	Yes ☐	No ☐
Gene	Yes ☐	No ☐
Chromosome	Yes ☐	No ☐
Genome	Yes ☐	No ☐
Genome sequencing	Yes ☐	No ☐

**Read the following questions and for each one answer True, False or Don’t know**			
	True	False	Don’t know
6. Our DNA is inside our cells	☐	☐	☐
7. Our DNA doesn’t have an effect on how our body works	☐	☐	☐
8. Our complete set of DNA is called our genome	☐	☐	☐
9. Around 1% of our genome is the same as other people’s	☐	☐	☐
10.Our genome is more similar to our close relatives, like our mum and dad, than it is with other people’s	☐	☐	☐
11.Genome sequencing involves looking at all the DNA in a person’s genome	☐	☐	☐
12. A ‘glitch’ in the genome (like a spelling mistake) can cause a health problem because the body isn’t getting the right instructions	☐	☐	☐
13.Genome sequencing can be done on the DNA in a blood sample	☐	☐	☐
14. Doctors and scientists know all there is to know about what our genome does	☐	☐	☐
15. If someone with a health problem has genome sequencing, they will always find helpful information about the cause of the problem	☐	☐	☐

QUESTIONNAIRE 2

Some questions about the animation

1. Was the animation easy or hard to understand?

☐ Very easy

☐ Quite easy

☐ Quite hard

☐ Very hard

2. The amount of information in the animation was:

☐ Too much

☐ Too little

☐ The right amount

3. The length of the animation was:

☐ Too long

☐ Too short

☐ The right amount

4. What did you think about the way the animation looked?

☐ I liked it very much

☐ I quite liked it

☐ I didn’t like it

5. Did you learn anything new?

☐ Yes

☐ No

☐ Not sure

6. Would you have found this animation helpful if you were making a decision about having genome sequencing?

☐ Yes

☐ No

☐ Don’t know

Some questions about your understanding

7. How would you describe your understanding of genetics?

☐ None

☐ Some

☐ Good

8. Have you heard of these words before?

DNA	Yes ☐	No ☐
Gene	Yes ☐	No ☐
Chromosome	Yes ☐	No ☐
Genome	Yes ☐	No ☐
Genome sequencing	Yes ☐	No ☐

9. Do you know what these words mean?

DNA	Yes ☐	No ☐
Gene	Yes ☐	No ☐
Chromosome	Yes ☐	No ☐
Genome	Yes ☐	No ☐
Genome sequencing	Yes ☐	No ☐

Read the following questions and for each one answer True, False or Don't know

	True	False	Don't know
10. Our DNA is inside our cells	☐	☐	☐
11. Our DNA doesn't have any effect on how our body works	☐	☐	☐
12. Our complete set of DNA is called our genome	☐	☐	☐
13. Around 1% of our genome is the same as other people's	☐	☐	☐

	True	False	Don't know
14. Our genome is more similar to our close relatives, like our mum and dad, than it is with other people's	☐	☐	☐
15.Genome sequencing involves looking at all the DNA in a person's genome	☐	☐	☐
16. A 'glitch' in the genome (like a spelling mistake) can cause a health problem because the body isn't getting the right instructions	☐	☐	☐
17. Genome sequencing can be done on the DNA in a blood sample	☐	☐	☐
18. We know all there is to know about what our genome does	☐	☐	☐
19. If someone with a health problem has genome sequencing, they will always find helpful information about the cause of the problem	☐	☐	☐

Some questions about your attitude to genome sequencing

20. Imagine you had a health problem and your doctor suggested genome sequencing to understand more about the cause of your condition. Would you want to have genome sequencing?

☐ Yes

☐ No

☐ Not sure

☐ I would want more information before I made a decision

21. I feel I understand the benefits of genome sequencing

☐ Agree

☐ Disagree

☐ Not sure

22. I feel I understand the limitations of genome sequencing (what it can't do)

☐ Agree

☐ Disagree

☐ Not sure

23. I feel the decision to have / not have genome sequencing would be easy for me to make

☐ Agree

☐ Disagree

☐ Not sure

Think about genome sequencing and answer the questions according to how you feel

24. Genome sequencing is:

☐ A bad thing

☐ A good thing

☐ Neither

25. Genome sequencing is:

☐ Harmful

☐ Helpful

☐ Neither

26. Genome sequencing is:

☐ Boring

☐ Interesting

☐ Neither

Finally, in your own words, let us know what you thought of the animation and if there is any other information you would have liked in it:

That is the end of the questionnaire. Thank you for taking part in this study!
