# Supplementary material for: Development and mixed-methods evaluation of an online animation for young people about genome sequencing
Source: Eur J Hum Genet. 2020 Jan 2;28(7):896–906. doi: 10.1038/s41431-019-0564-5 (PMC7316978; doi:10.1038/s41431-019-0564-5)
Supplement: Supplementary file 6 — Supplementary Material 6_Qualitative themes and supporting quotes [file 41431_2019_564_MOESM6_ESM.docx]

| Table 5: Qualitative themes and supporting quotes | | | |
| --- | --- | --- | --- |
|  | **Young people** | **Consenters** | **Parents** |
| **Theme 1:**  **The animation was an effective way of enhancing understanding about genome sequencing** | *“It made it better because I actually knew what they were talking about”….I’d have been thinking they were talking on another planet!” –* P8 female age 11  *“I thought it was pretty cool, because you could see what was going on”* – P4 male age 14  *“It wasn’t someone talking about it. It was clips showing it… It was a fun way of showing it”* – Sibling (female) of P2  *“I thought it was very explanative, like, it explained what the test was about and what happened during the test very well and about, like your DNA helps your body.”* P9 girl age 11 | *“It could help me gauge what they understood and what they knew and what they didn’t know, etc.”* – C2  *When I showed them this move, after the movie they said ‘oh yes, I have this question and this question and at this moment how long would it take for a result’ or things like that’* – C1  *“The second thing was some of the people were saying that they read the information sheet but they couldn't remember anything. So when I showed them the movie, a lot of them would tell me ‘oh yes I remember, that was nicely explained, etc.’ and then other people told me that the language was simple and it was much easier and nicer to have the information that way than reading long and lengthy forms.” -* C1 | *“I thought the video explained everything really well and I think that that was good for the younger age group and her age group because it was right across the pool. Very easy to understand”* – Mother of P6  *“The thing is, I had no idea. I had bits and pieces but they weren't, like, really getting through, but the way that they even showed how they were going to do the test and everything, which was quite good because now I know they're going to check the genes and how they're going to check it, so it wasn’t just saying to us ‘oh your genes are going to be checked’ and things like that. No, they were telling us how the four letters and if they're going to match and how they're not going to match, which was quite good, it was in detail, so I'm quite happy actually.”* - Mother of P5 |
| **Theme 2:**  **The animation helped young people feel more comfortable and engaged in the (research) process** | *“It introduces you to what you’re going to be doing and talk about it”-* P4 male age 14  *“Because, like, instead of, like, being nervous about the blood test and stuff, you could have it before so we could be less nervous instead…. Because we know why we’re taking the blood” –* P7 male age 11  *“Yeah, because now I know how it’s helping other people and me” –* P2 female age 11  *“If you watch the thing, I think it would make people, like, positive about what they’re doing and that it’s there to help them in the long run and that it may hurt for a bit and it may….and then it could help other people as well and I think that’s a good thing, so then they have a better mind going into it and it may take a bit of time but you might get a result and it might be good them or for other people.” –* P4 male age 14  *“Because before, I didn’t even know what it was and now I actually know what it is its making me more interested in my future”* – P8 girl age 11  *“I want to find out where all my genes came from and if they’ll still carry on” –* P8 girl age 11 | *“So most of the time they knew they were coming to the appointment to give blood because that’s the most stressful part of the project for anybody to take part…If someone was a bit nervous I don't think it took that away, but it definitely made them, it empowered them a little bit, so I know why I'm giving it, I don't like it, it might still hurt the same, but I know why I'm doing it.”* C1  *“I think it helped them to, like, understand why they were having a blood test”* C2  *“They were definitely more engaged and definitely felt more part of the consent process”* – C1  *“I found it quite a useful way to kind of getting engagement with the kids, because sometimes the consent discussion can be quite adult orientated and it ends up mostly being a discussion between me and the parents without so much involvement from children”* – C3  *“The research engagement was definitely heightened with this”* – C1 | *“She understands what’s going to happen, for the tests that she’s going to be doing” –* Mother of P5  *“And I think it was done in a way that kind of obviously made them feel a bit more at ease about it as well so it made us feel a bit more at ease about it”* - Father of P7  *“As a parent, if I had that video to start with, maybe [patient name] wouldn’t have had that little blip. It was the not knowing really.”* – Mother of P6  *“Yeah, I think the same, yeah. And, definitely, if I’d have seen that video, but I mean we’d already decided we was going to take part in the project, but if you was trying to look at appealing to a group of maybe children or parents that weren't sure, then using that video would definitely be the right way to do it.”* – Mother of P6 |
| **Theme 3: Showing the animation at the start of the appointment was most effective** | “At the beginning so you actually know what’s going to happen.” – P8 girl 11  *“I think it’s a good time because you can watch it and you can get told about it again if you don’t understand anything”* – P9 girl 11 | *“Mostly I chose to show it at the beginning of the sessions and I thought it was a nice icebreaker because it was something a little bit more informal than going into regulation and rights, etc.”* – C2 | *“Watching it here was much better. Much better, because it’s freshly done and we could ask questions”* Mother of P6 |
